# Supplementary material for: Diverse policy maker perspectives on the mental health of pregnant and parenting adolescent girls in Kenya: Considerations for comprehensive, adolescent-centered policies and programs
Source: PLOS Glob Public Health. 2023 Jun 20;3(6):e0000722. doi: 10.1371/journal.pgph.0000722 (PMC10281569; doi:10.1371/journal.pgph.0000722)
Supplement: Attachment — Submitted filename: revisionletterNov2022.docx [file pgph.0000722.s001.docx]

PGPH-D-22-00958R1	29^th^ November 2022
Diverse policy maker perspectives on the mental health of pregnant and parenting adolescent women in Kenya: Considerations for comprehensive, adolescent-centered policies and programs
Dr. Manasi Kumar

Dear Dr. Oliver Tolentino,

please find response to the comments and queries you made on the manuscript

1. Can you please upload a copy of your revised manuscript (clean copy) that does not contain any tracked changes, comments, or highlighting.
**Response**: we thank you and have done so now.

2. Please provide separate figure files in .tif or .eps format and remove any figures embedded in your manuscript file. Please also ensure that all files are under our size limit of 10MB.

For more information about how to convert your figure files please see our guidelines: https://journals.plos.org/globalpublichealth/s/figures

**Response**: please see edits made now, we have formatted the figures to .tif format, and removed them from the main manuscript.

3. Please amend your detailed online Financial Disclosure statement. This is published with the article. It must therefore be completed in full sentences and contain the exact wording you wish to be published.

a) Please clarify all sources of financial support for your study. List the grants, grant numbers, and organizations that funded your study, including funding received from your institution. Please note that suppliers of material support, including research materials, should be recognized in the Acknowledgements section rather than in the Financial Disclosure.

**Response**: We have provided this information in line 705- 709

b) State the initials, alongside each funding source, of each author to receive each grant. For example: "This work was supported by the National Institutes of Health (####### to AM; ###### to CJ) and the National Science Foundation (###### to AM)."

**Response**: We have indicated the initials, please see line 707

c) State what role the funders took in the study. If the funders had no role in your study, please state: “The funders had no role in study design, data collection and analysis, decision to publish, or preparation of the manuscript.”

**Response**: we have added - The funders had no role in study design, data collection and analysis, decision to publish, or preparation of the manuscript.

4. Please update your online Competing Interests statement. If you have no competing interests to declare, please state: “The authors have declared that no competing interests exist.”

**Response**: The authors have declared that no competing interests exist


5. We note that you have indicated that data from this study are available upon request from the authors. PLOS only allows data to be available upon request if there are legal or ethical restrictions on sharing data publicly. For more information on unacceptable data access restrictions, please see http://journals.plos.org/plosone/s/data-availability#loc-unacceptable-data-access-restrictions.

Before we proceed with your manuscript, please address the following prompts:

a) If there are ethical or legal restrictions on sharing a de-identified data set, please explain them in detail (e.g., data contain potentially identifying or sensitive patient information, data are owned by a third-party organization, etc.) and who has imposed them (e.g., a Research Ethics Committee or Institutional Review Board, etc.). Please also provide non-author contact information for a data access committee, ethics committee, or other institutional body to which data requests may be sent.

**Response**: the de-identified data can be shared from the corresponding author. The IRB committee can be contacted for further data sharing concerns using [uonknh_erc@uonbi.ac.ke](mailto:uonknh_erc@uonbi.ac.ke)

We will update your Data Availability statement on your behalf to reflect the information you provide.
